# Uranus: A Middleware Architecture for Dependable AAL and Vital Signs Monitoring Applications

**DOI:** 10.3390/s120303145

**Published:** 2012-03-07

**Authors:** Antonio Coronato

**Affiliations:** ICAR-CNR, Via P. Castellino 111, 80131 Naples, Italy; E-Mail: Antonio.Coronato@na.icar.cnr.it; Tel.: +39-81-613-9503; Fax: +39-81-613-9531

**Keywords:** vital signs monitoring, AAL, middleware services, rapid-prototyping, dependability

## Abstract

The design and realization of health monitoring applications has attracted the interest of large communities both from industry and academia. Several research challenges have been faced and issues tackled in order to realize effective applications for the management and monitoring of people with chronic diseases, people with disabilities, elderly people. However, there is a lack of efficient tools that enable rapid and possibly cheap realization of reliable health monitoring applications. The paper presents *Uranus*, a service oriented middleware architecture, which provides basic functions for the integration of different kinds of biomedical sensors. *Uranus* has also distinguishing characteristics like services for the run-time verification of the correctness of running applications and mechanisms for the recovery from failures. The paper concludes with two case studies as proof of concept.

## 1. Introduction

Ambient intelligence concerns a software system’s ability to be sensitive, adaptive, and responsive to changes in the physical environment [1]. AmI technologies are increasingly finding use in medical applications in locations such as hospitals and elderly homes, as witnessed by activities and communities such as [2–6], *etc*.

On the other hand, wearable sensor technologies have made remarkable improvements during the last decade and have attracted the interest of stakeholders from different domains like, as an example, healthcare. In this context, we have assisted in the creation of a new class of systems, the wearable eHealth systems. “*Wearable eHealth systems are convenient platforms for monitoring individual’s health-related parameters, even on a continuous basis, and for processing and feeding relevant information to their users and/or medical professionals*” [7].

A large number of monitoring systems, whose effectiveness and convenient economic impact have been widely demonstrated (e.g., [8]), have been realized for many diseases. Concerning, for example, cardiovascular diseases, which represent the leading cause of death worldwide, many wearable and portable eHealth systems have been developed (e.g., [9–11]). The non-invasive monitoring capability of these systems concerns not only the prevention of cardiovascular diseases (e.g., myocardial infarction and stroke), but also their management, as in the case of chronically ill patients.

In this scenario, however, despite a wide availability of technologies [12], there is still a lack of productivity tools to enable the production of applications ready for the market [13]. In addition to this, the need for more reliable, secure and adaptable solutions has also been emphasized [14]. Indeed, these applications must be considered safety critical and, consequently, should be designed with such a criticality taken into account. In [11], the authors have pointed out the weak points of their ECG tele-monitoring system from the point of view of the correctness. In the case of this application, the data must be processed in real-time; *i.e.*, by granting results within a certain temporal period. Moreover, in the case of critical values, alerts must be raised and processed by a temporal threshold. As stated by the authors, functional incorrectness or run time failures may result in catastrophic consequences for the patients monitored.

The methodology we developed to face such criticality is based on formal methods, which are well known techniques able to increase dependability by eliminating errors at the requirement specification and early design stages of the development [15]. We also use formal methods for runtime verification [16]; *i.e.*, the prototype system is monitored while it is running and checked against properties of interest (correctness properties) specified in a formal notation, so that whenever a violation of correctness properties is detected, a recovery strategy is triggered. In addition, traces of events related to failures are analyzed in order to isolate and remove faults.

This paper presents a middleware infrastructure, namely *Uranus*, which provides a set of basic services for the development of Ambient Assisted Living and vital signs monitoring applications. Two key points of *Uranus* are (1) the availability of mechanisms, tools and methodologies for the rapid prototyping of applications built on top of it and (2) the possibility of conferring stringent dependability requirements, which is another emerging issue in eHealth monitoring applications [17].

Definitively, the objective of this work is to provide a middleware infrastructure for the rapid prototyping of applications of Ambient Intelligence for healthcare, with a certain degree of dependability.

In this paper, Section 2 presents the architecture of *Uranus* and Section 3 introduces two case studies built on top of it. Section 4 describes major platforms available for the realization of vital signs monitoring applications. Next, Section 5 presents some evaluations and Section 6 reports our concluding remarks.

## 2. Uranus Architecture

**Figure 1.**
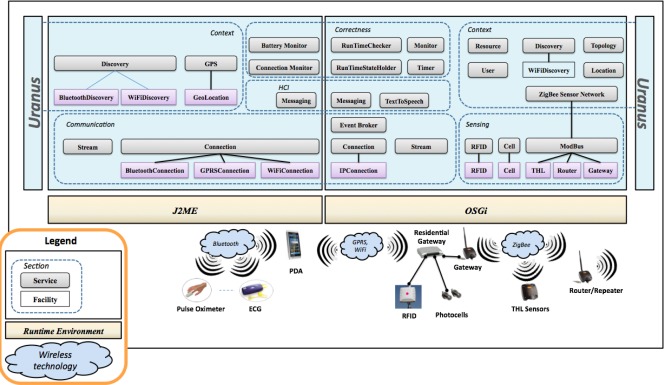
Uranus Architecture.

This section provides a description of both the architectural model of *Uranus* and its main services, which are depicted in Figure 1. The formal methodology supported by the middleware, as well as a preliminary version of some of the *Uranus*’ services of the middleware itself, has been presented in [18].

The infrastructure consists of two main packages of services and facilities, the first package provides mechanisms for the implementation of a residential gateway over the OSGi standard platform [19,20], the second one concerns software entities for mobile devices that support the J2ME environment.

A residential gateway is a component used to connect devices in the house to the Internet, and *vice-versa*. A home local area network may integrate environmental sensors and advanced human computer interface systems.

In *Uranus* the residential gateway is also the collecting point of sensed vital signs. Vital data are sensed by sensors and transmitted to a mobile device over a bluetooth network. Next, data are sent to the residential gateway by means of either a WiFi or a GPRS connection. The residential gateway can also catch environmental data sensed by zigbee sensors. Finally, it can be used to build a local monitoring station or to forward (some/all) sensed data towards a remote station deployed, for an example, in a hospital.

Current implementation integrates the resources described in Table 1.

In the following, we present the main components and logic packages of *Uranus*.

### 2.1. Environmental Sensing

This section concerns sensing parameters in a monitored environment. Three main kinds of environmental sensor are currently integrated in *Uranus*: (i) RFID short-range readers and (ii) photocell sensors, which are linked to the residential gateway, for the detection and identification of mobile objects and users; and (iii) THL ZigBee sensors for Temperature, Humidity and Lighting.

The section is structured in two different layers. The lowest layer integrates hardware devices. The heterogeneity of the components at this layer is handled by means of software entities called facilities, which hide hardware devices in the upper layer. Above this layer, a service handles the set of homogeneous facilities that stand below; *i.e.*, a facility service catches information coming from the set of facilities that are related to the same kind of hardware device. As an example, in the case of RFID sensors, *RFID Facilities* handle all the RFID antennas deployed in the environment; thus, each RFID antenna is controlled by its own facility that manages interactions between the hardware component and the rest of the software system. Above this layer, the *RFID Service* collects information coming from all the *RFID Facilities* (even for different kinds of antennas) and provides such information to the rest of the services after a preliminary processing. In this way, the replacement of underlying hardware technologies affects only the facility layer with a limited impact on the overall system.

Other facilities and services of this section are those for handling a ZigBee WSN that senses environmental parameters such as temperature, humidity and lighting (e.g., *THL*, *Router*, *gateway* and *ModBus*) and those for detecting the presence of people in certain areas of the monitored environment (*i.e.*, *Photocells*).

### 2.2. Communication

This section offers communication functionalities. In particular, an asynchronous communication mechanism is provided by the *Event Service*. This service supports the publish-subscribe mechanism [21] and is in charge of dispatching events between software components. Other communication services concern the opening of network connections (*Connection Service*) and the handling of data streams (*Stream Service*). Currently, *Uranus* handles three kinds of network connection: WiFi, GPRS, and Bluetooth.

### 2.3. Human Computer Interaction

This section includes mechanisms for natural interactions in a smart environment. Indeed, one of the great challenges for future AmI applications is to make interactions with technology-rich environments intuitive, natural, unobtrusive, intelligent, and distraction free. Currently, such a section provides services like *TextToSpeech*—to synthesize vocal messages—and *Messaging*—to send textual messages to the user’s mobile device.

### 2.4. Context

This section concerns the handling of context information. It includes the following services and facilities: *Topology Service*, which provides a unique and uniform representation for the topology of the environment. In particular, locations are classified as semantic locations—buildings, floors, rooms, specific parts of a room (e.g., the area in front of a wall monitor)—and physical locations—the area sensed by an RFID antenna, the area covered by a WiFi access point, *etc*. [22]; *Location Service*, which is in charge of handling physical location information for mobile resources and users. In particular, it locates mobile users and resources tagged with RFIDs orWiFi enabled devices [22] indoors. An outdoor location service is granted for mobile devices equipped with a Global Positioning System and the *GPS Service; ZigBee Wireless Sensor Network Service*, which receives environmental parameters, such as temperature, humidity, and lighting, from a ZigBee sensor network; *User Service* that provides basic authentication mechanisms for users and handles the list of all the people in the environment; *Resource Service*, which supports mechanisms for registering and monitoring resources. In addition, it allows an association between users and resources (e.g., an oximeter attached to a specific patient); and, *Discovery Service* that supports functionalities for the automatic detection of devices connected either to a WLAN or to a Bluetooth network.

### 2.5. Correctness

The last section concerns mechanisms for the runtime verification of correctness properties specified by means of a formal methodology that we defined in [23].

*Uranus* provides a set of generic services defined for runtime verification, which consists of the *RunTimeStateHolder Service*, (this service holds and exposes the state of an ambient, as intended in *Ambient Calculus*), the *RunTimeChecker Service* (this service checks, on behalf of an ambient, the correctness properties), the *Timer Service* (this service holds and verifies temporal constraints), and the *Monitoring Service* (this service monitors the entire system). Moreover, two specific monitors have been defined for mobile devices. The *Battery Monitor* checks the level of the battery of the mobile device. The *Connection Monitor*, instead, handles connection handover and switches.

## 3. Case Studies

This section presents two case studies developed on top of *Uranus*. The first concerns an application for the long-term monitoring of vital signs, whereas the second consists of an application for a reliable smart hospital that includes the short term monitoring of vital signs and some context-aware features.

### 3.1. Long-Term Monitoring

This application has been realized for the long term (e.g., for 48 h) monitoring of the oxygen in the blood of a chronically ill patient. A residential gateway is deployed at the home of the patient, although the monitoring must continue even when the patient is at work or elsewhere outside the home. This rises the need of handling implicit requirements like the power consumption of battery driven devices, network switching, and reliability assurance.

The system includes an oximeter, permanently attached to the patient, which senses the value of the oxygen and transmits it to a PDA. The PDA, in turn, forwards data to the residential gateway. Data are transmitted either over the WiFi domestic network while the patient is at home, or over the GPRS network otherwise.

The system must be able to detect connection failures when the patient leaves the house; *i.e.*, it must switch from the WiFi connection to the GPRS connection. On the contrary, when the patient come back home, the system must reuse the WiFi domestic connection.

Another important issue concerns the power consumption of battery driven devices, which is a limiting factor for long-term monitoring. In spite of the emergence of new technologies [24] and new standards like the bluetooth low energy profile, this issue can not be considered definitively solved [25]. For this reason, the system must be able to detect low battery levels and to migrate onto spare devices.

**Figure 2.**
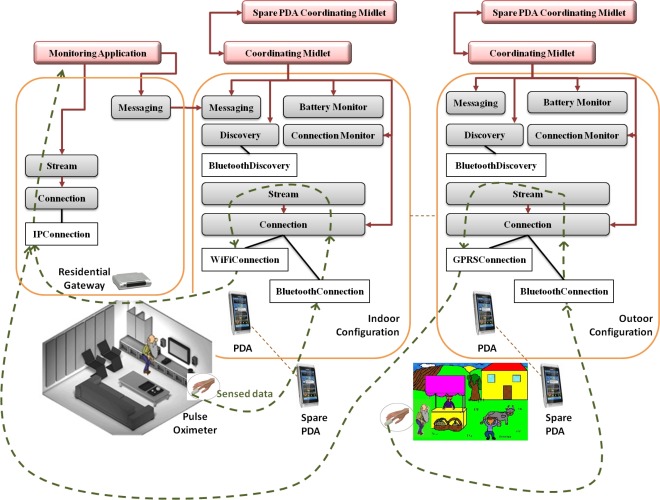
Case Study 1.

The system architecture is shown in Figure 2.

The PDA receives data sensed by the oximeter through a *Bluetooth Connection* facility. Next, the PDA’s *Stream Service* transmits the data to the Residential Gateway’s *Stream Service* either through a *WiFi Connection*, while the patient is at home, or a *GPRS Connection* if the patient is elsewhere. Finally, the data stream is received and analyzed by a *Monitoring Application* built on top of the residential gateway.

The *Connection Monitor* surveys the availability of the domestic WiFi. In particular, in the case of the patient leaving home, the *Connection Monitor* detects the WiFi connection failure and requires the *Connection Service* to start a GPRS connection. In contrast, when the patient comes back home, the *Connection Monitor* reveals the availability of the domestic network and imparts the *Connection Service* to which from the *GPRS Connection* to a *WiFi Connection*.

Another issue concerns the power consumption of the PDA. We can assume that the patient is equipped with one (or even more) spare PDA. When the level of the battery of the primary PDA reaches a certain threshold, the *Battery Monitor* alerts the *Coordinating Midlet*, which sends a message through the Messaging Service to require the spare PDA be turned on. Next, the two coordinating midlets start a coordination protocol. In particular, they discover each other by means of the *Discovery Service*. Then, the primary PDA releases its bluetooth and WiFi connections, while the spare PDA starts to handle the data stream.

**Figure 3.**
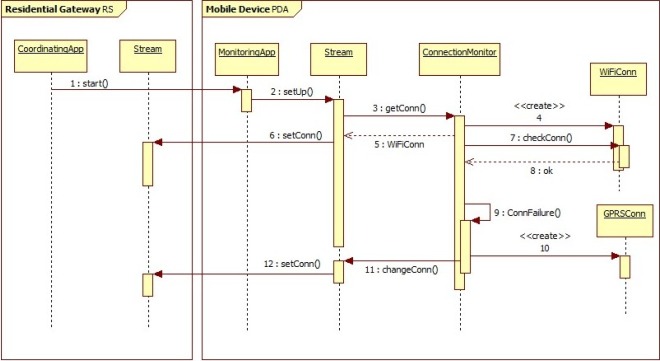
Component Interactions.

Figure 3 presents some interactions between software components of the first case study. In particular, interactions show how the application can self-reorganize after the failure of a network connection. Indeed, the *ConnectionMonitor*, which periodically checks for the availability of the connection, detects the failure (maybe due to fact that the patient has left the home) and activates a new available connection.

It is worth noting that in order to realize this application only two new components have been implemented, the *Monitoring Application* and the *Coordinating Midlet*. All the other services have been imported from *Uranus* without any modification.

### 3.2. Smart Hospital

The system presented is an application of ambient intelligence for a department of Nuclear Medicine.

Within the department, the patients are injected with a small amount of a radioactive substance in order to undergo specific examinations (e.g., Blood Volume Study, Bone Scan, Brain Scan, *etc*.). Once injected, patients emit radiation (gamma rays) and have to stay in a specific room to wait for the examination to be performed. The time the patients have to wait depends on the kind of examination that has to be performed and the time the radioactive substance takes to propagate—within the body—and to decay to the right level. In fact, examinations can be executed only if the radiation level is within a certain range. After the examination, the patients return to the waiting room until the level of radiation falls below a specific threshold and so becomes harmless.

Currently, the patients are accompanied by nurses when moving within the department. The goal is to have an automatic system that would guide them within the department and monitor their heart rate to identify possible undesired effects of the injection.

The department consists of four semantic locations: (1) the *Acceptance Room* (AR), which is the room where the patients are accepted within the department and wait for the injection; (2) the *Injection Room* (IR), which is the room where the patients receive the injection; (3) the *HotWaiting Room* (HWR), which is the room where the patients wait for the examination after having been injected and until the radiation level reaches the correct range; and (4) *Diagnostic Room* (DR), which is the room where examinations are performed. The last three rooms are equipped with short-range RFID readers.

**Figure 4.**
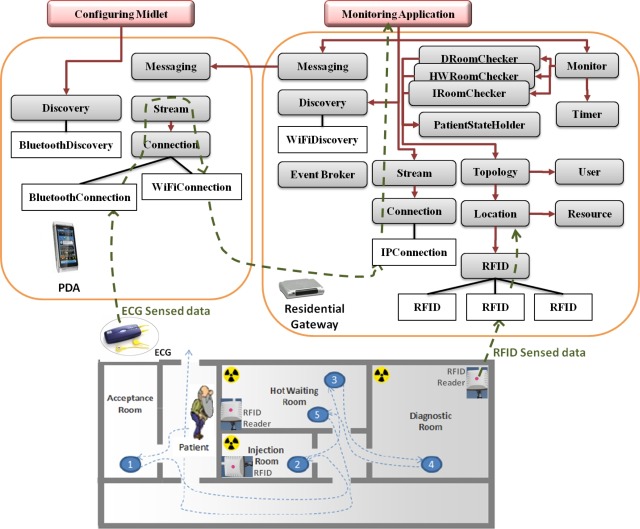
Case Study 2.

The architecture of the system prototype is partly described in Figure 4.

Within the acceptance room, an operator registers the patient with the *User Service* and equips him with an RFID tag, an ECG sensor, and a PDA. The resources are rapidly configured by means of services like the *Discovery Service* and applications like the *Configuring Midlet*, and are associated to the user by means of the *Resource Service*. From this moment, the patient’s heart rate is monitored and the data stream is handled as in the previous case study.

In addition to this, when the patient moves in the radioactive area, the *Location Service* determines the presence of a new RFID tag in a physical location by means of the *RFID Service*; e.g., an event like {Tag = 127; RFID Reader = 2} is produced. Such an event is translated into semantic information (e.g., {Patient = ‘Massimo Rossi’; Location = ‘Injection Room’}) by means of the *Topology Service*, which handles a description of the topology of the department, and the *Resource Service*, which allows an identification of the patient corresponding to the RFID tag.

Every time the patient changes his position within the department, several correctness properties are verified by the *Uranus* correctness services. In fact, several correctness properties are formally specified using *Ambient Calculus* and *Ambient Logic* (the reader can refer to [23] for a more detailed description of the formal models realized for this case study).

The patient’s status is maintained by the *PatientStateHolder Service* and is verified by the *HotWaitingRoomChecker Service* whenever the patient attempts to enter the hot waiting room. In the case of the patient trying to enter a location at an incorrect time (*i.e.*, his status is different from that which allows him to be admitted), he will be required to leave the room and to move in the correct one by means of a message sent by the *Messaging Service*.

As for the hot waiting room, similar constraints have been specified and are verified by the *DiagnosticRoomChecker* and the *InjectionRoomChecker* when the patient tries to enter the diagnostic room or the injection room respectively.

The *Timer*, instead, alerts the *Monitor Service* whenever a temporal threshold is overcome.

In order to implement such a prototype, two specific components have been realized, the *Configuring Midlet* and the *Monitoring Application*. Among the *Uranus* services, those concerning the correctness properties (*PatientStateHolder*, *HotWaitingRoomChecker*, *InjectionRoomChecker*, *DiagnosticRoom-Checker*, and *Timer*) have been customized for the case study. All the other services have been imported from *Uranus* without any modification.

**Figure 5.**
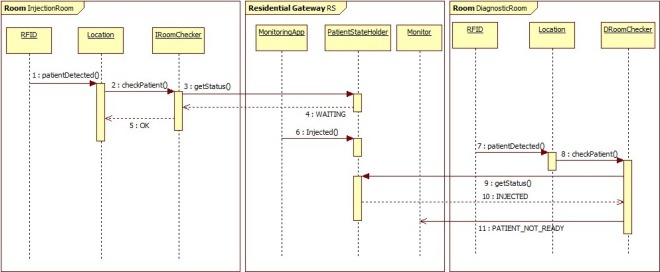
Component Interactions.

Figure 5 shows some of the interactions between a subset of software components into the department. In particular, after having been located into the injection room, the monitoring application updates the patient status. Next, the patient enters the diagnostic room by mistake. As a consequence, a correctness properties is violated and the diagnostic room checker alerts the monitor service. Successively (not shown in the figure), a message is sent to the patient’s mobile device and to the staff of the diagnostic room.

## 4. Related Work

This section presents a couple of examples of existing systems and recent enabling platforms, whose main characteristics are reported in Table 2, for the monitoring of patient’s vital signs.

Among many examples of such system, we report *WEALTHY* [26] and *LOBIN* [27]. *WEALTHY* is a health monitoring system based on a textile wearable interface implemented by integrating sensors, electrodes, and connections in a fabric form, advanced signal processing techniques, and telecommunication systems. Sensors, electrodes and connections are realized with conductive and piezoresistive yarns.

*LOBIN*, instead, is a system for healthcare monitoring in hospital environments. The main features of this system are the capability of monitoring different kinds of vital signs due to the possibility of the integration of several kinds of (even commercial) ZigBee sensors and the availability of some location facilities within the hospital.

Concerning the enabling platforms for the realization of monitoring applications, the most remarkable examples (to the best of our knowledge) are here reported.

*SYLPH* [28] is a service oriented architecture for the integration of different kinds of WSN (e.g., Bluetooth and Zigbee). The platform is shown to be useful in the case of home monitoring that requires the perception and analysis of both human vital signs and environmental parameters. *SYLPH* does not provide outdoor monitoring facilities or indoor location awareness mechanisms.

*MaRV* [29] is a multi-agent platform, which is able to handle a large set of wireless sensor technologies. In addition to standard wireless technologies, it also handles NFC (near field communication) [30] enabled devices. *MaRV* has been mainly conceived as a tool to improve the assistance to patients in a geriatric department and provides some motion analysis and location awareness facilities within the hospital.

In [31] Junnila *et al.* present a general purpose home area sensor network and monitoring platform. The monitoring platform is multi-purpose because the system is easily configurable for various user needs and is easy to set up. It consists of a chosen set of sensors, a wireless sensor network, a home client, and a distant server. however, it is not available for free, but, instead, it can be rented by organizations or individuals.

*Mercury* [32] is an open source wireless sensor platform specifically devised for the motion analysis of patients being treated for neuromotor disorders, such as Parkinson’s Disease, epilepsy, and stroke. *Mercury* is designed to support long-term, longitudinal data collection for patients in hospital and home settings. It also presents characteristics that assure dependability to its applications like reliable data streaming, energy consumption monitoring and sensor heartbeats. It is important to note that, although *Mercury* is an open source project, several functionalities rely on the Harvard “Pluto” mote, which is not available on the market.

*SPINE* [33] (Signal Processing in Node Environment) is another open-source framework for the development of Body Sensor Network (BSN) applications. It provides developers of signal processing algorithms with APIs and libraries of protocols, utilities and data processing functions. In particular, it offers advanced functions for behavior analysis.

*Uranus* is a more general service oriented middleware architecture that provides facilities for the realization of a large set of Ambient Assisted Living and vital signs monitoring applications. *Uranus* is an open source project available at [34]. In *Uranus*, monitoring is performed both indoors and outdoors since it is a platform able to handle and switch between different kinds of connections (*i.e.*, WiFi and GPRS). It also supports methodologies and provides mechanisms for the dependability of monitoring applications. Indeed, the design is supported by a formal methodology [23] and the platform provides mechanisms like runtime verification of correctness properties, monitoring of the energy consumption of mobile devices and self-reconfiguring that confer a certain degree of reliability. Finally, applications based on *Uranus* can be partly designed and configured by a visual tool, namely *Ambient Designer* [35], which both supports the formal methodology and provides visual models and testing. Another relevant facility offered by *Ambient Designer* for the rapid prototyping of new applications is the ability to autoconfigure *Uranus*’s services from visual models realized during the design stage. *Ambient Designer* is also an open source project available at [36].

Definitively, *Uranus* presents the following relevant (sometimes exclusive) characteristics: (i) it is open-source; (ii) it is part of a specifically devised formal methodology; (iii) it offers several mechanisms for the dependability of its applications; and (iv) it is supported by a visual modeling and testing tool for the rapid prototyping.

## 5. Discussion

This section presents some considerations concerning the usefulness and effectiveness of *Uranus*.

First, it is possible to evaluate the level of reuse of *Uranus* services in order to realize the two case studies. As reported in Table 3, 80% of the software line of codes (LOC) of the first case study have been obtained from *Uranus* without any modification. The rest of the LOCs have been written from scratch. For the second case study, 74% of the software has been obtained from *Uranus*, 21% written from scratch and the remaining 5% obtained by specializing/rewriting *Uranus* artifacts. Specifically, this last one regards the runtime verification services.

Secondly, some results on the dependability of the two case studies can be deduced. We focus on the fault coverage of the runtime verification mechanisms. Let us start with the first case study (Table 4). We consider four types of faults: *Battery low power*, *WiFi disconnection*, *Sensed data not delivered on time*, and *Sensed data corrupted*. Concerning the first two types of faults, the system, being equipped with battery and connection monitors along with additional logic for coordinating the spare PDA, is able both to detect and recover the failure. In addition, if the system is equipped with a timer (also available in Uranus) for monitoring the delay and jitter of transmitted data, it will also be able to detect excessive delay in the transmission of vital signs. However, with the current architecture there is no mechanism for recovering from this failure.

Concerning the second case study, two classes of fault have been taken into account: human faults and system faults. Human faults are caused by an incorrect—either intentional or unintentional—behavior of the system user. As far as correctness properties that can be expressed in terms of the constructs reported in Section 3.3 (e.g., *canEnter*, *canLeave*, *canInvoke*, and *canWrite*, *etc*., in the case that they are combined with time constraints) are concerned, both the modeling of the constraints and the runtime verification are quite easy. Many kinds of human fault in AmI can be detected by means of such properties. A few examples are reported in Table 5. In contrast, the detection and recovery of some kinds of system fault may be more difficult. As an example, an *RFID missed read*, which occurs when an RFID tag is not read by an antenna, is not detected by the system of constraints defined for the case study; whereas, an *RFID cross read*, which takes place when an RFID tag is read contemporaneously by two or more antennas, can be detected but not recovered because the runtime monitor is unable to distinguish the correct location. Additionally, a message delivery failure can be detected and recovered with a system of constraints and timers; whereas, the proposed mechanisms cannot help to detect content corruption. To achieve this aim, other mechanisms (e.g., error correction code) must be integrated.

To conclude, the proposed approach is suitable for correctness properties that are related to the physical location of users, mobile resources and code. Concerning such kinds of constraint, the methodology provides mechanisms for both the specification and runtime verification.

## 6. Conclusions

Vital signs monitoring is a field of application that has been receiving great attention from several kinds of stakeholder, all interested in the realization of systems and applications which are effective, reliable, economically convenient, and capable of improving the quality of any National Healthcare Service by reducing hospitalizations and assuring a better quality of life for patients. The possibility of having a large class of monitoring applications—reliable and economically convenient—ready for the market is still a target far from being achieved.

This paper has presented a middleware infrastructure, *Uranus*, which is open source and is aimed at (i) providing mechanisms for a generic class of monitoring application; (ii) assuring a high degree of reliability for the applications built on top of it, supporting a formal designing methodology and runtime verification mechanisms for correctness; and (iii) easing the development of and supporting some visual design and auto-configuring features.

## Figures and Tables

**Table 1.** HW Resources.

**Device**	**Producer**	**Model**
*PDA*	Nokia	N8
*ECG Sensor*	Alive Tech. Pty.	Alive ECG
*Oximeter*	Alive Tech. Pty.	Alive Pulse Oxim.
*Zigbee THL Sensor*	4-Nocks	ZED-THL-M
*Zigbee Gateway*	4-Nocks	ZC-GW-USB-EM
*Zigbee Router*	4-Nocks	ZR-REP-EM
*Short-Range RFID*	FEIG	OBID LRU2000
*Photocells*	Omron Ind. Aut.	M18 cylindrical

**Table 2.** The Main Characteristics of Systems and Platforms.

**System**	**Architecture**	**Wireless Technologies**	**Monitoring**	**Data-Analysis**	**Location Awareness**	**Dependability**	**RAD**	**Open Source**
*WEALTHY*	Embedded	-GPRS	-Indoor -Outdoor	-Simple alarm generation				
*LOBIN*	Embedded	-ZigBee	-Indoor	-Simple alarm generation	-Indoor (ZigBee-based)			
*SYLPH*	SOA	-ZigBee -BlueTooth -WiFi	-Indoor					
*MaRV*	Multi-Agent	-ZigBee -NFC -WiFi -GPRS -RFID	-Indoor	-Motion analysis	-Indoor (RFID-Based)			
*Junnila et al.*	Embedded	-ZigBee	-Indoor	-Behavior analysis	-Indoor (Floar’s Sensor-Based)			
*Mercury*	Component-based	-ZigBee	-Indoor	-Advanced Motion streaming analysis		-Reliable data -Sensors heartbeat -Energy consumption monitoring		*X*
*SPINE*	Component-based	-ZigBee	-Indoor	-Advanced Behavior analysis -Simple alarm generation				*X*
*Uranus*	SOA	-ZigBee -BlueTooth -WiFi -GPRS -RFID	-Indoor -Outdoor	-Simple alarm generation	-Indoor (RFID-Based) -Outdoor (GPS-Based)	-Formal design -Runtime verification mechanisms -Energy consumption monitoring -Self-reconfiguring	-Visual modeling and testing	*X*

**Table 3.** Reuse of *Uranus* Services for the Two Case Studies.

**Metric**	**Case Study 1**	**Case Study 2**
*LOC of the system*	≈2,000	≈8,900
*LOC from* Uranus	≈1,600 (80%)	≈6,600 (74%)
*LOC of* Uranus *adapted*	0	≈500 (5%)
*LOC brand new*	≈400 (20%)	≈1,800 (21%)

**Table 4.** Example of Fault Coverage for Case Study 1.

**Fault**	**Detection**	**Recovery**
*Battery low power*	*X*	*X*
*WiFi disconnection*	*X*	*X*
*Sensed data not delivered on time*	*X*	
*Sensed data corrupted*		

**Table 5.** Example of fault coverage for Case Study 2.

**Fault type**	**Fault**	**Detection**	**Recovery**
Human Fault	Patient enters a wrong location	*X*	*X*
	Radioactive substance timeout	*X*	*X*
	Medical operator calls a wrong patient	*X*	*X*
	Medical operator sets up a wrong patient status	*X*	*X*

System Fault	Message undelivered to the patient’s mobile device	*X*	*X*
	Message content corrupted		
	RFID cross read	*X*	
	RFID missed read		
